# She Never Had a Period: A Case of Mayer-Rokitansky-Küster-Hauser Syndrome

**DOI:** 10.7759/cureus.116675

**Published:** 2026-09-21

**Authors:** Aya Saber, Tarek Rammah

**Affiliations:** 1 Pediatrics, Internal Medicine, Mercy Medical Care, Brooklyn, USA

**Keywords:** adolescent, no period, pelvic mri, primary amenorrhea, uterine agenesis

## Abstract

Primary amenorrhea is a common concern in adolescent females and can place significant emotional pressure on both the patient and her parents. Although often attributed to benign causes, it may reflect underlying congenital anomalies even in the presence of normal pubertal development. We report the case of a 15-year-old girl of Middle Eastern descent who presented with absence of menarche. She reported normal pubertal development and denied disordered eating or excessive exercise. Hormonal evaluation demonstrated follicle-stimulating hormone and estrogen levels within normal limits. Pelvic magnetic resonance imaging revealed non-visualization of the uterus with normal-appearing ovaries, findings later confirmed by pelvic ultrasonography. Brain magnetic resonance imaging demonstrated an unremarkable pituitary gland. These findings led to the diagnosis of Mayer-Rokitansky-Küster-Hauser syndrome. This case underscores the importance of structural imaging in the evaluation of primary amenorrhea, even when hormonal studies and pubertal development are normal.

## Introduction

Primary amenorrhea is a frequent reason for referral during adolescence and is often initially attributed to constitutional delay, particularly when pubertal development appears normal [1,2]. However, congenital structural anomalies of the reproductive tract may present with few outward signs and normal hormonal profiles, increasing the risk of delayed or missed diagnosis [2,3]. Primary amenorrhea is defined as the absence of menarche by age 15 in individuals with normal pubertal development, or by age 13 in those without pubertal development [1,2]. The differential diagnosis is broad, ranging from constitutional delay to androgen insensitivity syndrome, and includes hormonal, congenital, lifestyle-related, and nutritional causes [1,2]. This condition often remains undetected until adolescence, when menstruation fails to occur [3,4].

Mayer-Rokitansky-Küster-Hauser (MRKH) syndrome is a congenital condition resulting from failure of Müllerian duct development [3,5,6]. It is characterized by the absence of the uterus and variable absence of the upper vagina in individuals with normal ovarian function and secondary sexual characteristics [3,5]. This case highlights the importance of early imaging in adolescents presenting with primary amenorrhea, even when initial laboratory evaluation is reassuring.

## Case presentation

A 15-year-old girl of Middle Eastern descent presented to the clinic with her parent due to concern regarding the absence of menarche. She reported normal pubertal development, including breast development and pubic hair growth beginning at age 12. She denied cyclic pelvic pain, galactorrhea, excessive exercise, disordered eating, or chronic medical illness. She had never been sexually active. Her blood pressure was consistently normal throughout all visits, and serum sodium and other electrolytes in her metabolic panel were within normal limits.

Family history was notable for siblings with congenital heart disease and autism spectrum disorder. There was no known family history of reproductive tract anomalies or disorders of sexual development.

Initial evaluation focused on common endocrine causes of primary amenorrhea. Hormonal testing demonstrated follicle-stimulating hormone and estrogen levels within normal limits (Table 1). Given the reassuring hormonal profile and normal pubertal development, further investigation with imaging was pursued.

**Table 1:** Serum estrogen and follicle-stimulating hormone (FSH) levels

Name	Value	Reference Value
Estrogen, total, serum	531.0 (pg/ml)	Follicular phase (90-590), Luteal phase (130-460)
FSH	3.38 (MIU/ML)	Follicular phase (3.85-8.78), Mid-Cyclic peak (4.54-22.51), Luteal phase (1.79-5.12)

Pelvic magnetic resonance imaging demonstrated non-visualization of the uterus with normal-appearing ovaries, including bilateral ovarian follicles and a right ovarian corpus luteum. A pelvic ultrasound performed several months later confirmed these findings (Figure 1). Based on the imaging results, a diagnosis of MRKH syndrome was established. Brain magnetic resonance imaging was also obtained and demonstrated a normal-sized pituitary gland without a definite focal lesion (Figure 2).

**Figure 1:**
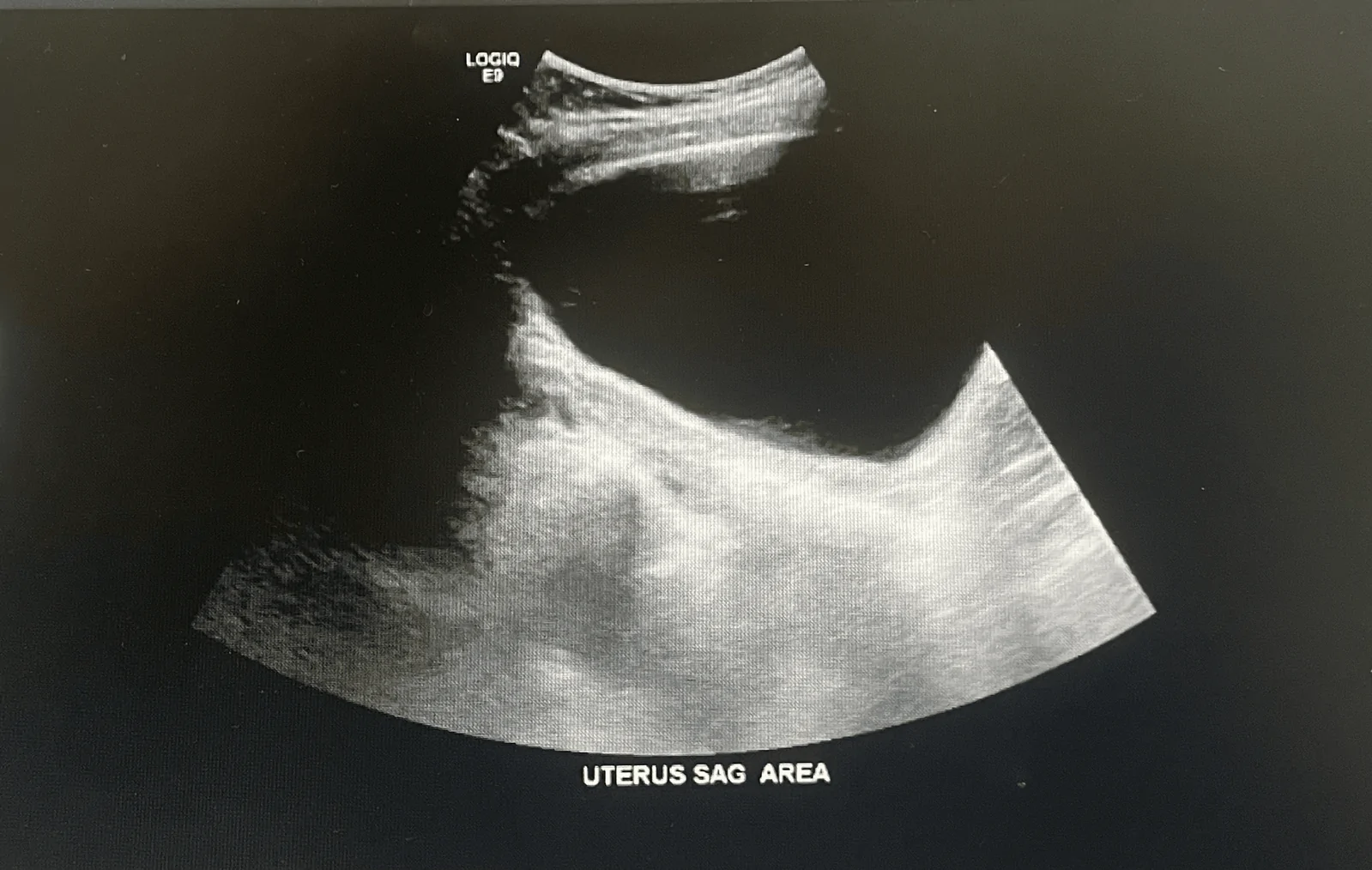
Pelvic ultrasound image The image demonstrates the expected uterine sagittal region, with no uterus visualized.

**Figure 2:**
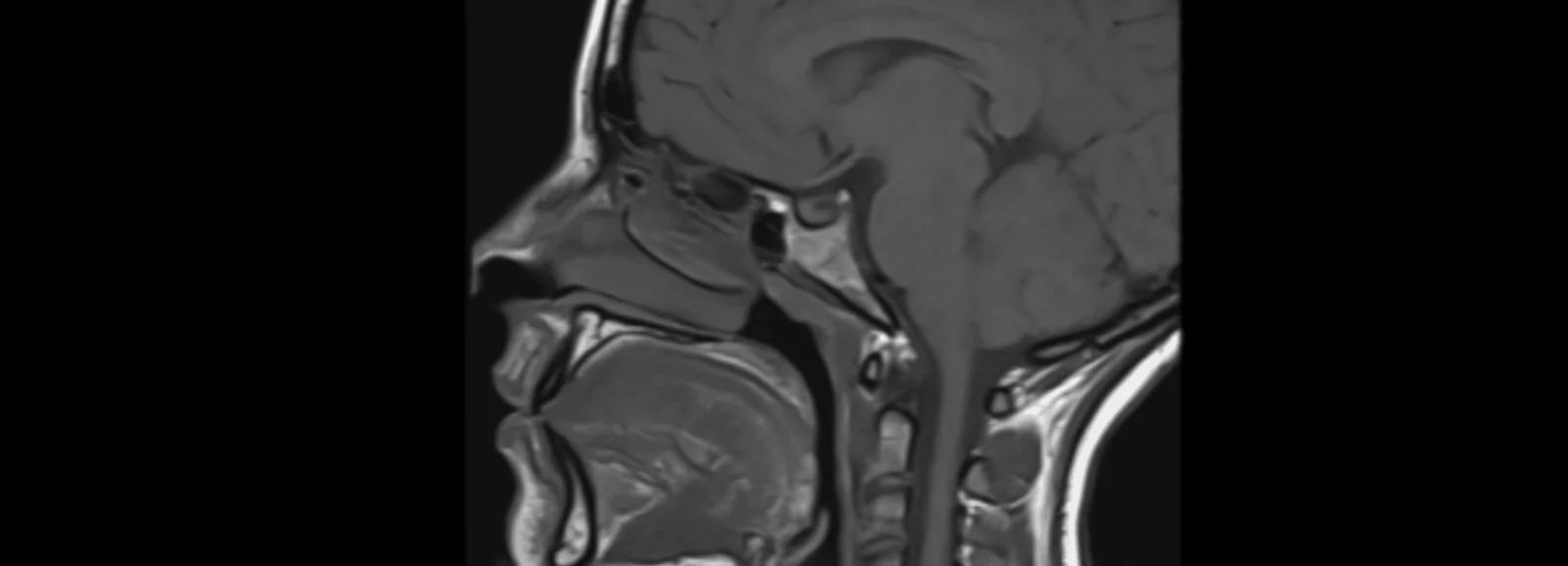
Brain MRI The pituitary gland is normal in size, with no detectable lesions.

The diagnosis was discussed in detail with the patient and her parents at the time it was established. Counseling focused on the congenital nature of the condition and explained the absence of menstruation despite normal pubertal development. It was emphasized that the condition was unrelated to hormonal imbalance, lifestyle factors, or delayed puberty. Anticipated reproductive implications were addressed with sensitivity, and the patient was referred to a gynecologist and a pediatric endocrinologist for further evaluation and counseling. Genetic testing for the patient and siblings was discussed with the guardian. A vaginal examination for the patient was deferred for cultural reasons.

## Discussion

MRKH syndrome affects approximately 1 in 4,500 individuals assigned female at birth and is most commonly diagnosed during adolescence following evaluation for primary amenorrhea [3,4]. Because ovarian function is preserved, affected individuals develop normal secondary sexual characteristics, which can delay recognition of the condition and contribute to diagnostic uncertainty [3,5].

This case illustrates how normal hormonal findings may provide false reassurance and delay definitive diagnosis unless structural imaging, such as pelvic ultrasound or MRI, is performed [1-3]. Early use of pelvic imaging is therefore essential in adolescents presenting with primary amenorrhea, even when endocrine evaluation is normal [2,3]. Notably, pelvic imaging not only established the diagnosis but also helped exclude other differential diagnoses, such as androgen insensitivity syndrome, by confirming normal ovarian structure and function [1,2].

The presence of congenital conditions among the patient’s siblings raises consideration of shared developmental or genetic factors. However, MRKH syndrome is most often sporadic and multifactorial [5,6]. Further research is needed to better understand potential familial associations.

Beyond its anatomical features, MRKH syndrome carries significant psychosocial implications. Adolescents diagnosed with MRKH may experience distress related to infertility, body image, and future family planning [7]. Early diagnosis, clear communication, and involvement of both the patient and family are critical for supporting psychological well-being and long-term adjustment [7]. Long-term follow-up in this patient indicates that she has maintained normal development and has come to understand and accept her diagnosis, highlighting the value of early counseling and supportive care.

## Conclusions

Primary amenorrhea warrants comprehensive evaluation regardless of normal pubertal development and reassuring hormonal studies. Mayer-Rokitansky-Küster-Hauser syndrome, while rare, has lifelong reproductive and psychosocial implications. Early recognition, appropriate imaging, and thoughtful counseling can reduce uncertainty and help patients and families understand and navigate a diagnosis that fundamentally alters reproductive expectations.
